# The effect of a decaffeinated green tea extract formula on fat oxidation, body composition and exercise performance

**DOI:** 10.1186/s12970-014-0062-7

**Published:** 2015-01-21

**Authors:** Justin D Roberts, Michael G Roberts, Michael D Tarpey, Jack C Weekes, Clare H Thomas

**Affiliations:** Department of Life Sciences, Anglia Ruskin University, East Road, Cambridge, UK; School of Life & Medical Sciences, University of Hertfordshire, College Lane, Hatfield, Hertfordshire UK

**Keywords:** Green tea, Body composition, Fat oxidation, Exercise performance

## Abstract

**Background:**

The cardio-metabolic and antioxidant health benefits of caffeinated green tea (GT) relate to its catechin polyphenol content. Less is known about decaffeinated extracts, particularly in combination with exercise. The aim of this study was therefore to determine whether a decaffeinated green tea extract (dGTE) positively influenced fat oxidation, body composition and exercise performance in recreationally active participants.

**Methods:**

Fourteen, recreationally active males participated in a double-blind, placebo-controlled, parallel design intervention (mean ± SE; age = 21.4 ± 0.3 yrs; weight = 76.37 ± 1.73 kg; body fat = 16.84 ± 0.97%, peak oxygen consumption [$\dot{\mathrm{V}}{\mathrm{O}}_{2\mathrm{peak}}$] = 3.00 ± 0.10 L·min^−1^). Participants were randomly assigned capsulated dGTE (571 mg·d^−1^; n = 7) or placebo (PL; n = 7) for 4 weeks. Following body composition and resting cardiovascular measures, participants cycled for 1 hour at 50% $\dot{\mathrm{V}}{\mathrm{O}}_{2\mathrm{peak}}$, followed by a 40 minute performance trial at week 0, 2 and 4. Fat and carbohydrate oxidation was assessed via indirect calorimetry. Pre-post exercise blood samples were collected for determination of total fatty acids (TFA). Distance covered (km) and average power output (W) were assessed as exercise performance criteria.

**Results:**

Total fat oxidation rates increased by 24.9% from 0.241 ± 0.025 to 0.301 ± 0.009 g·min^−1^ with dGTE (*P* = 0.05; ηp^2^ = 0.45) by week 4, whereas substrate utilisation was unaltered with PL. Body fat significantly decreased with dGTE by 1.63 ± 0.16% in contrast to PL over the intervention period (*P* < 0.001; ηp^2^ = 0.84). No significant changes for FFA or blood pressure between groups were observed. dGTE resulted in a 10.9% improvement in performance distance covered from 20.23 ± 0.54 km to 22.43 ± 0.40 km by week 4 (*P* < 0.001; ηp^2^ = 0.85).

**Conclusions:**

A 4 week dGTE intervention favourably enhanced substrate utilisation and subsequent performance indices, but did not alter TFA concentrations in comparison to PL. The results support the use of catechin polyphenols from dGTE in combination with exercise training in recreationally active volunteers.

## Introduction

The health benefits of polyphenols found in green tea (GT), the unfermented leaves of the tea plant, *Camellia sinensis*, have been extensively investigated in the last fifteen years [1-7]. Studies have demonstrated antioxidant [8,9] and chemoprotective properties [4], as well as improvements in cardio-metabolic health from various GT strategies (including reduced circulating cholesterol and triglyerides [10], increased thermogenesis and whole body fat oxidation [1,3,11], reduced blood pressure [7,12,13] and improved body mass index ratios [5,14-17]). These health benefits, in part, relate to the bioactive catechin polyphenol content of GT, of which (−)-epigallocatechin-3-gallate (EGCG) can account for between 50–80% of the total catechin content [18].

GT catechins have been proposed to influence metabolic and thermogenic activities in the short term, via inhibition of catechol-o-methyl transferase (COMT) leading to enhanced catecholamine, cAMP and lipolytic activity [17,19,20], although this has been disputed [20]. GT catechins, particularly EGCG, may also activate endothelial nitric oxide synthase, leading to mild reductions in blood pressure [13,21].

In the longer term, GT catechins may influence specific signalling molecules, including PGC1α, leading to gene expression of fat metabolism enzymes [20]. Whilst such mechanisms are currently under debate, strategies to enhance fat oxidation, body composition and cardiovascular efficiency in conjunction with physical activity are of pertinence to the general population. Additionally the indirect sparing of glycogen stores may support improved exercise tolerance and/or performance.

Research investigating GT extracts (GTE) and exercise have produced conflicting results. Modest EGCG dosage in the short term (270 mg·d^−1^ EGCG for 6 days [22], and 68 mg·d^−1^ EGCG for 3 weeks [23,24]) did not alter metabolic or performance variables in healthy or endurance trained volunteers. However, the inclusion of 100.5 mg·d^−1^ EGCG over a 10 week training period enhanced whole-body metabolic efficiency elsewhere [11]. One confounding factor though is the use of caffeinated GTE in these studies. When decaffeinated GTE (dGTE) has been employed, 366 mg EGCG was found to acutely increase fat oxidation by 17% [3]. Conversely, higher dosage dGTE (624 ± 3 mg·d^−1^ EGCG for 28 days) did not significantly affect fat oxidation in healthy, male volunteers [25].

We were therefore invited to undertake an independent assessment of the cardio-metabolic and performance effects of a dGTE formula (571 mg·d^−1^ GTE providing 400 mg·d^−1^ EGCG) over a 4 week period in comparison to placebo in healthy, male volunteers. It was hypothesised that moderate dose dGTE would significantly improve fat oxidation and performance, supporting longer term mechanisms linking GTE catechins to enhanced metabolic enzyme gene expression.

## Materials and methods

### Participants

Fourteen healthy, male participants volunteered following power calculation assessment (G*Power3, Dusseldorf [26]; using α = 0.05; 1-β = 0.95; based on observed data [3,22-24] and 2 groups). Participants were required to be recreationally active non-smokers, and have no known sensitivities to tea products or be regular green tea consumers. Prior to study inclusion, all participants provided written informed consent and satisfactorily completed a general health screen. The study was approved by the University of Hertfordshire Life and Medical Sciences Ethics Committee. Participant characteristics are displayed in Table 1.

**Table 1:** **Baseline characteristics and resting measurements across the intervention**

**Variable**	**PL (n = 7)**			**dGTE (n = 7)**		
Age (years)	21.4 ± 0.6			21.4 ± 0.3		
Height (m)	1.77 ± 0.03			1.78 ± 0.01		
$\dot{\mathrm{V}}{\mathrm{O}}_{2\mathrm{peak}}$ (L·min^−1^)	3.13 ± 0.18			2.87 ± 0.08		
	**Baseline**	**Week 2**	**Week 4**	**Baseline**	**Week 2**	**Week 4**
Weight (kg)	75.46 ± 2.91	75.11 ± 2.94*	74.81 ± 2.88*	77.29 ± 2.05	76.96 ± 2.03*	76.69 ± 1.95* ^B^
Bodyfat (%)	16.63 ± 1.58	16.34 ± 1.69	15.97 ± 1.69*^#^	17.06 ± 1.24	16.23 ± 1.39*	15.43 ± 1.33*^# A,B^
HR (bpm)	61.00 ± 2.70	62.14 ± 1.81	61.00 ± 1.83	62.00 ± 1.25	62.43 ± 2.01	62.57 ± 1.92
SBP (mm Hg)	129.29 ± 1.73	125.71 ± 3.64	127.00 ± 2.44	130.00 ± 1.18	126.57 ± 2.14	126.86 ± 3.64
DBP (mm Hg)	67.57 ± 4.35	66.14 ± 1.72	69.71 ± 2.37	69.57 ± 2.40	65.71 ± 2.55	67.71 ± 2.11

Table 1 shows the key participant characteristics for each group, including absolute changes for weight, body fat, heart rate and blood pressure over the intervention. Data are presented as mean ± SE. PL, Placebo; dGTE, decaffeinated green tea extract; $\dot{\mathrm{V}}{\mathrm{O}}_{2\mathrm{peak}}$, peak oxygen uptake; HR, heart rate; SBP, systolic blood pressure; DBP, diastolic blood pressure. ^A^denotes significant overall group x time interaction effect (*P* = 0.002). ^B^denotes significant overall time interaction effect only (*P* < 0.001). ^*^denotes significant difference (*P* ≤ 0.05) to baseline only within group. ^#^denotes significant difference to week 2 within group only (*P* < 0.046).

### Procedures

#### Preliminary testing

All testing took place in the Human Physiology Laboratory, University of Hertfordshire. Participants were instructed to refrain from consuming caffeinated products for 48 hours prior to initial testing, and not be consuming other supplementation.

Peak oxygen consumption ($\dot{\mathrm{V}}{\mathrm{O}}_{2\mathrm{peak}}$) was assessed at least one week prior to experimental trials using a standard incremental step protocol increasing by 30 W each 3 minutes until volitional exhaustion as previously reported [27]. Tests were performed on a Monark Ergomedic 874E stationary bike (Monark Exercise AB, Varberg, Sweden) using a Metalyser 3B automated gas-analyser (Cortex Biophysik, Leipzig, Germany). On a separate occasion, subjects undertook a familiarisation trial to confirm exercise intensity at 50% $\dot{\mathrm{V}}{\mathrm{O}}_{2\mathrm{peak}}$. This intensity was selected based on pilot work in which average fat oxidation rates during sustained submaximal exercise were statistically greater at 50% $\dot{\mathrm{V}}{\mathrm{O}}_{2\mathrm{peak}}$ compared to both 40 and 60% $\dot{\mathrm{V}}{\mathrm{O}}_{2\mathrm{peak}}$.

#### Treatments

Participants were randomly assigned to an experimental or placebo group, and provided with either capsulated dGTE (571 mg·d^−1^ dGTE, delivering 70% or 400 mg·d^−1^ EGCG (equivalent to 6–7 cups of green tea per day), Changsha Active Ingredients Group Inc., Changsha, China*), or placebo (700 mg·d^−1^ corn flour). All participants received capsules on a weekly basis to monitor compliance, with instructions to consume one capsule daily before breakfast with 250 ml water. *Analysis of the main active ingredient (EGCG) was undertaken prior to and independently of the main study by Changsha Active Ingredients Group Inc., using high performance liquid chromatography. The certificate of analysis provided by the supplying company demonstrated that the product contained 91.21% total catechins, from which 70.74% was EGCG. The product did not appear to be assessed for other catechins, so it is likely that the remaining percentage comprised other catechins (GCG, EGC, GC, EC, ECG, etc.).

#### Experimental design and intervention

A randomised, double blind, placebo controlled parallel design was employed over a 4 week intervention. Participants completed three laboratory trials at week 0, 2 and 4 under controlled conditions following an overnight fast. Upon arrival, nude body mass (Seca 780, Hamburg, Germany), height (Seca 200 stadiometer, Hamburg, Germany) and body composition [28] (Tanita Body Segmental Analyser 418-BC, Tokyo, Japan) were assessed.

Participants were then fitted with a Polar FS2c telemetric monitor (Polar Electro Ltd., Kempele, Finland) and seated for 5 minutes prior to resting heart rate and blood pressure readings (Omron MX3 plus, Kyoto, Japan). A venous wholeblood sample was then collected into duplicate 4 ml K_3_EDTA Vacutainers (Greiner Bio-One GmbH, Kremsmunster, Austria) by a qualified phlebotomist. Samples were centrifuged for 10 minutes at 2000 rpm, with aliquotted plasma immediately frozen at −80°C for later assessment of TFA.

#### Exercise trials

Exercise trials comprised a submaximal assessment and performance stage. During the submaximal assessment, participants exercised for one hour at 50% $\dot{\mathrm{V}}$O_2peak_ on a Monark Ergomedic 874E cycle ergometer. Gas exchange data was recorded continuously throughout exercise using a Metalyser 3B gas analyser, with average data taken over the final 45 minutes of submaximal exercise. Rating of perceived exertion (RPE) [29] and heart rate were recorded every 20 minutes.

Rates of total carbohydrate oxidation (CHO_TOT_), total fat oxidation (FAT_TOT_) (g·min^−1^) and energy expenditure (EE) (kJ·min^−1^) were calculated from $\dot{\mathrm{V}}{\mathrm{O}}_2$ and $\dot{\mathrm{V}}{\mathrm{CO}}_2$ (L·min^−1^) using stoichiometric equations [30], with protein oxidation assumed negligible, as follows:

1 $$ {\mathrm{CHO}}_{\mathrm{TOT}}=4.210\bullet \left({\dot{\mathrm{V}}\mathrm{C}\mathrm{O}}_2\right)\hbox{--} 2.962\bullet \left({\dot{\mathrm{V}}\mathrm{O}}_2\right) $$

2 $$ {\mathrm{FAT}}_{\mathrm{TOT}}=1.695\bullet \left({\dot{\mathrm{V}}\mathrm{O}}_2\right)\hbox{--} 1.701\bullet \left({\dot{\mathrm{V}}\mathrm{C}\mathrm{O}}_2\right) $$

3 $$ \mathrm{E}\mathrm{E}=\left[\left(0.550\bullet \dot{\mathrm{V}}{\mathrm{CO}}_2\right)\hbox{--} \left(4.471\bullet \dot{\mathrm{V}}{\mathrm{O}}_2\right)\right]\bullet 4.2 $$

Upon completion, seated blood pressure and post exercise venous sampling was repeated as previously described. Following this, participants were instructed to undertake a 40 minute self-paced performance trial using a Computrainer erogometer system (RaceMate Inc., Seattle, USA). Distance covered (km) and power output (W) were recorded each 10 minutes, with only time elapsed visible to the subjects. Verbal encouragement was provided each 10 minutes. At the end of the exercise trial, subjects recovered for 5 minutes at 50 W.

#### Dietary intake and exercise activity

All participants recorded a 3 day dietary recall preceding each exercise trial to assess for habitual dietary compliance. Dietary analyses were undertaken using Dietplan 6.50 (Forestfield Software Ltd, West Sussex, United Kingdom), with no differences reported between groups for macronutrients and/or energy intake. Additionally, participants were requested to consume similar meals the day before the exercise trials at regular time intervals to provide increased control of oxidation variables. This was based on pilot work assessment of fat oxidation stability at 50% $\dot{\mathrm{V}}{\mathrm{O}}_{2\mathrm{peak}}$ (assessed by calculating the amount of time (minutes) throughout the exercise that was spent within ±0.02 g·min^−1^ of the average fat oxidation rate, expressed as a percentage for each individual), which was greater when the 24 hour pre exercise period was controlled for dietary intake (68.22 ± 5.70%) compared to when only the evening meal preceding the testing session was controlled (60.78 ± 8.42%).

The standardised menu was based on typical foods consumed by participants at breakfast, lunch and dinner, and provided similar caloric intake to habitual dietary records (values as calorie totals and per kilogram mean bodyweight: energy intake: 2484.70 kcal·d^−1^ (32.68 kcal·kg^−1^·d^−1^); carbohydrate: 1127.7 kcal·d^−1^ (14.83 kcal·kg^−1^·d^−1^); fat: 565.4 kcal·d^−1^ (7.43 kcal·kg^−1^·d^−1^) and protein: 791.96 kcal·d^−1^ (10.42 kcal·kg^−1^·d^−1^). Throughout the intervention period, participants were instructed to minimise consumption of polyphenol rich foods. Participants were additionally required to cycle for one hour at 50% $\dot{\mathrm{V}}{\mathrm{O}}_{2\mathrm{peak}}$ three times per week as part of a regulated exercise programme. All participants provided training diaries to monitor compliance.

#### Blood analyses

All blood analyses for TFA were independently undertaken by ABS Laboratories (Biopark, Welwyn Garden City, Hertfordshire) employing previously validated methods [31]. TFA concentrations were based on assessment of palmitic, palmitoleic, stearic, oleic and linoleic acids. Briefly, 100 μl plasma aliquots were spiked with internal standard (heptadecanoic acid), with free fatty acids being extracted using the ‘Dole Extraction Solvent’ (isopropanol/heptane/sulphuric acid (1 M) (40:10:1)). After drying under nitrogen, the extracts were re-suspended using 200 μL of dichoromethane and the FFAs derivatised using diethylamine and Deoxo-Fluor. The diethylamide derivatives were then extracted into heptane. The heptane was then removed using nitrogen in a dry-block at 70°C. The dried extracts were reconstituted into 100 μL of heptane and quantified by gas chromatography using a mass spectrometer as the detector in the selected ion monitoring (SIM) mode.

#### Statistical analyses

Statistical analyses were performed using SPSS (v19, Chicago, USA). Baseline variables were assessed using an independent samples *t*-test. A mixed design repeated measures analysis of variance (ANOVA) was employed to assess treatment and time interactions. Where pertinent, a one way ANOVA with Bonferroni post hoc adjustments was utilised to assess within treatment effects. An alpha level of ≤0.05 was employed for statistical significance. Data are reported as means ± SE.

## Results

### Baseline characteristics and resting measures

Intervention groups were matched for age, height, weight, body fat and $\dot{\mathrm{V}}{\mathrm{O}}_{2\mathrm{peak}}$ at baseline (Table 1). A significant interaction effect for bodyweight was found across time only (F = 16.98, *P <* 0.001). Net bodyweight reduction was similar between groups across the intervention (0.64 ± 0.17 kg for PL; F = 12.33, *P* = 0.001; ηp^2^ = 0.67), and 0.60 ± 0.21 kg for dGTE; F = 6.27, *P* = 0.014; ηp^2^ = 0.51 within group). There was a significant group x time interaction for percentage body fat (F = 7.81, *P* = 0.002), with an overall reduction of 1.63 ± 0.16% with dGTE (*P* < 0.001; ηp^2^ = 0.84 within group) compared to 0.66 ± 0.15% for PL (*P* = 0.002; ηp^2^ = 0.66 within group). No significant effects were reported for resting heart rate or blood pressure.

### Submaximal exercise measures

**Figure 1:**
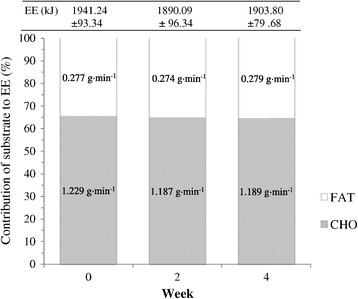
**Weekly contribution of substrate to total energy expenditure (EE) for the PL group.** Figure 1 shows the contribution of both fat and carbohydrate (based on oxidation rates) to energy expenditure during submaximal exercise for the placebo condition. Data are presented as mean ± SE. PL, Placebo; FAT, average fat oxidation rates; CHO, average carbohydrate oxidation rates. No significant differences were found with ANOVA (*P* > 0.05).

**Figure 2:**
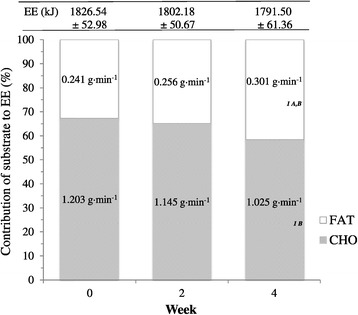
**Weekly contribution of substrate to total energy expenditure (EE) for the dGTE group.** Figure 2 shows the contribution of both fat and carbohydrate (based on oxidation rates) to energy expenditure during submaximal exercise for the dGTE condition. Data are presented as mean ± SE. dGTE, decaffeinated green tea extract; FAT, average fat oxidation rates; CHO, average carbohydrate oxidation rates. ^A^denotes significant overall group × time interaction effect compared with PL (Figure 1; *P* = 0.05). ^B^denotes significant overall time interaction effect in conjunction with PL (Figure 1; *P* ≤ 0.03). ^1^ denotes significant interaction over time within GTE only (*P* ≤ 0.05).

Weekly contribution of substrate to total energy expenditure (EE) for PL and dGTE are reported in Figures 1 and 2 respectively. No significant differences were reported for EE either between or within groups over time (*P* > 0.05), demonstrating consistency of the submaximal exercise trials.

A significant overall group x time interaction for FAT_TOT_ was observed (F = 3.39, *P* = 0.05). FAT_TOT_ during exercise remained similar for PL across the intervention period (week 0 = 0.277 ± 0.038 g·min^−1^; week 2 = 0.274 ± 0.031 g·min^−1^; week 4 = 0.279 ± 0.030 g·min^−1^, *P* > 0.05) despite a non-significant increase in percentage contribution to total EE (week 0 = 34.61 ± 3.72%; week 2 = 35.23 ± 2.99%; week 4 = 35.53 ± 2.53%, *P* > 0.05).

FAT_TOT_ for dGTE increased from 0.241 ± 0.025 g·min^−1^ at week 0 to 0.256 ± 0.023 g·min^−1^ at week 2, and to 0.301 ± 0.009 g·min^−1^ by week 4 (F = 4.10, *P* =0.05; ηp^2^ = 0.45). This represented a 24.9% or 0.060 ± 0.027 g·min^−1^ increase in FAT_TOT_ with dGTE. Correspondingly, percentage contribution of total fat to exercise EE increased with dGTE from 32.61 ± 3.53% at week 0 to 34.71 ± 2.57% at week 2, and to 41.45 ± 1.31% at week 4 (F = 4.28, *P* = 0.045; ηp^2^ = 0.46).

A significant time interaction was observed only for CHO_TOT_ (F = 4.28, *P* = 0.028). CHO_TOT_ reduced with dGTE from 1.203 ± 0.078 g·min^−1^ at week 0, to 1.144 ± 0.044 g·min^−1^ at week 2, and finally to 1.025 ± 0.048 g·min^−1^ by week 4, representing a reduction of 14.8% or 0.178 ± 0.069 g·min^−1^ (F = 4.02, *P* = 0.05; ηp^2^ = 0.45).

Correspondingly, percentage contribution of total CHO to exercise EE also reduced with dGTE from 67.39 ± 3.53% at week 0 to 65.28 ± 2.57% at week 2, and to 58.55 ± 1.31% at week 4 (F = 4.28, *P* = 0.045; ηp^2^ = 0.46). CHO_TOT_ and contribution of CHO to EE were largely unaffected with PL (*P* > 0.05).

**Figure 3:**
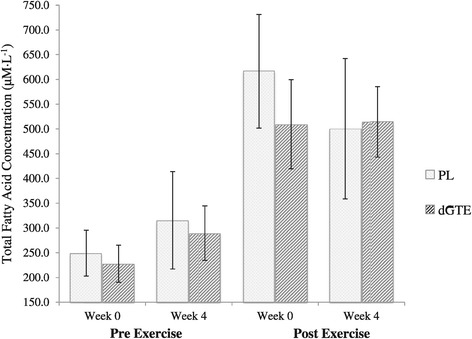
**Total fatty acid concentrations pre and post exercise.** Figure 3 shows the absolute total fatty acid concentrations at rest and post exercise for both treatment conditions at week 0 and week 4. Data are presented as mean ± SE. PL, Placebo; dGTE, decaffeinated green tea extract. No significant differences were found with ANOVA (*P* > 0.05).

Despite an improvement in FAT_TOT_ for dGTE, no significant differences were observed for TFA concentrations either within or between groups pre-post intervention (Figure 3, *P* > 0.05). It was however noted that TFA concentrations were elevated pre-exercise by week 4 with PL (249.3 ± 46.2 μM·L^−1^ at week 0 to 315.4 ± 98.1 μM·L^−1^ at week 4, a 26.5% increase, *P* > 0.05) and dGTE (227.9 ± 37.6 μM·L^−1^ at week 0 to 289.7 ± 54.8 μM·L^−1^ at week 4, a 27.1% increase, *P* > 0.05), with lack of significance most likely explained by individual variance.

It was also noted that whereas TFA concentrations reduced by 18.8% post exercise in the PL group from 616.5 ± 114.8 μM·L^−1^ at week 0 to 500.3 ± 141.8 μM·L^−1^ at week 4; post exercise TFA concentrations were maintained with dGTE (509.3 ± 90.0 μM·L^−1^ at week 0 to 514.3 ± 71.1 μM·L^−1^ at week 4), although no significant differences were reported within or between groups (*P* > 0.05). No significant differences were reported for any of the individual fatty acids measured.

Submaximal oxygen uptake values were not different across time either within or between groups (Table 2) demonstrating compliance to the set intensity (*P* > 0.05). No significant interaction effects were found for expired carbon dioxide, despite a modest reduction in $\dot{\mathrm{V}}{\mathrm{CO}}_2$ during submaximal exercise with dGTE from 1.31 ± 0.05 L·min^−1^ at week 0, to 1.25 ± 0.05 L·min^−1^ at week 4 (*P* > 0.05). However, in conjunction with improved FAT_TOT_, a significant overall group × time interaction was observed for the respiratory exchange ratio (RER; F = 3.30, *P* = 0.05), with values reducing from 0.90 ± 0.01 at week 0 to 0.87 ± 0.01 at week 4 (F = 4.36, *P* = 0.044; ηp^2^ = 0.47, within group) supporting reduced reliance on CHO with dGTE. No such modifications for RER were observed with PL (*P* > 0.05).

**Table 2:** **Assessment of oxygen uptake, mean heart rate, perceived exertion and blood pressure related to submaximal exercise**

	**PL (n = 7)**			**dGTE (n = 7)**		
**Variable**	**Week 0**	**Week 2**	**Week 4**	**Week 0**	**Week 2**	**Week 4**
$\dot{\mathrm{V}}{\mathrm{O}}_2$ (L·min^−1^)	1.55 ± 0.08	1.51 ± 0.08	1.52 ± 0.06	1.46 ± 0.04	1.44 ± 0.04	1.44 ± 0.05
$\dot{\mathrm{V}}{\mathrm{CO}}_2$ (L·min^−1^)	1.38 ± 0.06	1.35 ± 0.06	1.35 ± 0.05	1.31 ± 0.05	1.29 ± 0.03	1.25 ± 0.05
RER	0.89 ± 0.01	0.89 ± 0.01	0.89 ± 0.01	0.90 ± 0.01	0.89 ± 0.01	0.87 ± 0.01 ^1 A,B^
HR (b·min^−1^)	127.8 ± 5.5	122.7 ± 4.4	121.5 ± 3.8	124.9 ± 3.7	117.0 ± 2.5	113.9 ± 4.0 ^1 B^
RPE (6–20)	11.1 ± 0.8	11.7 ± 0.6	11.6 ± 0.3	11.9 ± 0.4	11.2 ± 0.4	10.0 ± 0.6* ^A^
SBP (mm Hg)	132.7 ± 2.9	127.4 ± 3.0	127.7 ± 2.9	132.3 ± 2.4	127.6 ± 1.8	126.3 ± 2.9
DBP (mm Hg)	80.1 ± 3.3	74.7 ± 1.9	73.0 ± 2.0	74.4 ± 1.8	76.6 ± 5.1	70.1 ± 3.5

Table 2 demonstrates the influence of the dGTE on cardio-respiratory measures during submaximal steady state exercise across the intervention. Data are presented as mean ± SE. PL, Placebo; dGTE, decaffeinated green tea extract; $\dot{\mathrm{V}}{\mathrm{O}}_2$, submaximal oxygen uptake; $\dot{\mathrm{V}}{\mathrm{CO}}_2$, submaximal expired carbon dioxide; RER, respiratory exchange ratio; HR, heart rate; RPE, rating of perceived exertion; SBP, systolic blood pressure; DBP, diastolic blood pressure. ^A^denotes significant overall group x time interaction effect (*P* ≤ 0.05). ^B^denotes significant overall time interaction effect only (*P* ≤ 0.02). ^1^denotes significant within group time interaction effect only (*P* ≤ 0.045). ^*^denotes significant difference within group to baseline (*P* = 0.015).

Although submaximal exercise heart rate reduced by 6.24 ± 3.85 b·min^−1^ (4.8% by week 4) in the PL group, significance across time was only found with dGTE where submaximal exercise heart rate reduced by 8.8% from 124.95 ± 3.69 b·min^−1^ at week 0 to 113.90 ± 4.03 b·min^−1^ at week 4 (F = 4.07, *P* = 0.045; ηp^2^ = 0.40). A significant overall group × time interaction was also found for RPE (F = 3.43, *P* = 0.05), with subjects perceiving exercise to be progressively easier with dGTE by week 4 (10.0 ± 0.6 relative effort rating) compared to week 0 (11.9 ± 0.4; *P* = 0.015; ηp^2^ = 0.58). No differences were reported for systolic or diastolic blood pressure immediately post exercise over time for either group (*P* > 0.05).

#### Performance measures

**Figure 4:**
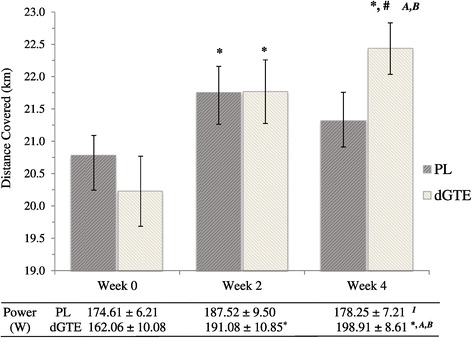
**Distance covered and average power output during the performance trial.** Figure 4 shows the distance covered and average power output elicited during the 40 minute performance trial for both treatment conditions at week 0, 2 and 4 of the intervention. Data are presented as mean ± SE. PL, Placebo; dGTE, decaffeinated green tea extract. ^A^denotes significant overall group × time interaction effect (*P* ≤ 0.001). ^B^denotes significant overall time interaction effect (*P* < 0.001). ^1^denotes significant within group time interaction effect only (*P* = 0.039). ^*^denotes significant difference (*P* ≤ 0.02) to baseline only within group. ^#^denotes significant difference to week 2 within group only (*P* = 0.03).

A significant overall group × time interaction was found for distance covered (F = 9.84, *P* = 0.001; Figure 4). The use of dGTE resulted in a progressive increase in distance covered from 20.23 ± 0.54 km at week 0, to 21.77 ± 0.49 km at week 2 and finally 22.43 ± 0.40 km by week 4, representing a 10.9% significant increase in performance (F = 28.66, *P* < 0.001; ηp^2^ = 0.85). A similar interaction effect was also observed for PL (F = 7.94, *P* = 0.009; ηp^2^ = 0.61) with distance covered significantly improving by week 2 (21.75 ± 0.40 km) compared to week 0 (20.79 ± 0.30 km; *P* = 0.002) only.

In a similar manner, a significant overall group × time interaction was found for average power output (F = 14.43, *P* < 0.001; Figure 4). Average power output increased with dGTE (F = 40.01, *P* < 0.001; ηp^2^ = 0.89) by 17.9% or 29.02 ± 5.53 W from week 0 (162.06 ± 10.08 W) to week 2 (191.08 ± 10.85 W; *P* = 0.01); and by 22.7% (or 36.85 ± 3.20 W) from week 0 to week 4 (198.91 ± 8.61 W; *P* <0.001), but was not significantly different between week 2 and 4 (*P* > 0.05). No significant differences across time for average power output were observed for PL (*P* > 0.05).

## Discussion

The use of a 4 week dGTE strategy significantly enhanced FAT_TOT_ by 24.9% or 0.060 ± 0.027 g·min^−1^ compared to PL supporting the hypothesis set. The increased contribution of total fat to EE with dGTE supports the proposal that EGCG positively influences substrate utilisation, particularly in combination with exercise training. The use of a capsulated dGTE formula in the present study potentially offers a more practical means to regularly consume a sufficient daily dosage to elicit such effects (compared to consumption of ~6-7 cups of green tea per day, especially considering the notable variability of catechin content in commercial green teas).

Improvements in FAT_TOT_ have been demonstrated elsewhere [1,11,32], with current values comparable to those reported when employing an acute dGTE strategy with similar EGCG content [3]. Conversely, higher dose dGTE over a 28 day period did not enhance substrate metabolism in healthy, male volunteers [25] in contrast to these findings. However, in this latter study, the higher FAT_TOT_ are more typical of endurance trained athletes. It has been inferred that the combined effect of exercise training and GTE may be more relevant for untrained individuals who ‘respond’ to GTE intervention [25]. Participants in this study were recreationally active. It is therefore plausible that adaptations in exercise metabolism with dGTE are more pronounced with less trained individuals, as opposed to physically active or endurance trained volunteers assessed elsewhere [23-25].

Improved FAT_TOT_, and reduced reliance on CHO_TOT_ during exercise are of clinical and performance relevance. Although resting and post exercise TFAs were not significantly different between groups, the increase in FAT_TOT_ with dGTE supports the contention that the inhibition of COMT may not be a dominant mechanism.

Independently of antioxidant protective mechanisms, it has been proposed that EGCG positively modulates cell signalling via PGC1α [20], sirtuin 1 (SIRT1) and mitogen activated protein kinase (MAPK) pathways [33,34]. In the longer term (>4 weeks), it is feasible that EGCG at moderate dose facilitates up-regulation of gene expression leading to enhanced FAT_TOT_ with exercise.

Although bodyweight reductions were similar between groups, the use of dGTE combined with regulated exercise significantly reduced body fat compared to PL. These findings are similar to those reported elsewhere [10,32,35], particularly when either higher dose GTE or low dose caffeine has been employed. Alterations in body composition, coupled with increased FAT_TOT_ infer that catechin polyphenols favourably modulate cellular metabolism, possibly via a calorie restriction mimetic (CRM) action [36]. This contention is further supported via studies demonstrating enhanced glucose tolerance, insulin sensitivity and adiponectin levels with EGCG [3,37].

In the current study, resting and submaximal exercise heart rate and blood pressure decreased over the intervention period in both groups. However, results were only significant over time with dGTE for exercising heart rate and perceived exertion, possibly relating to substrate utilisation efficiency and improved exercise economy. Additionally, whilst the results could also indicate an acute training stimulus, non-significant reductions in SBP found were comparable to those observed elsewhere [10,13,37]. It is therefore suggested that any mild hypotensive effects are likely due to the short-term influence of regular aerobic activity on nitric oxide pathways than dGTE impacting on endothelial production of nitric oxide synthase.

There has been much interest in the use of GTE to enhance physical performance. In animal studies, time to exhaustion has been shown to improve with GTE by 8-24%, with corresponding evidence of increased ß-oxidation and fatty acid translocase/CD36 mRNA expression [38]. When relatively low GTE/EGCG doses have been employed in humans, improvements in time trial or performance measures have not been observed [22,24]. However, the inclusion of matched caffeine placebo or pre-exercise feeding may explain these findings. Conversely, with higher dose GTE strategies, improvements in maximal oxygen uptake and time trial performance have been observed [39,40].

To the authors’ knowledge, this is the first study to demonstrate a significant impact of dGTE on subsequent exercise performance. Performance indices improved by 10.9% for distance covered, and 22.7% for average power output with dGTE. This is unlikely to be fully explained via a training effect as improvements with PL were only observed at week 2 of the trial. Reduced reliance on CHO_TOT_ may have contributed to improved performance following submaximal exercise.

Future research investigating specific effects of EGCC from dGTE on exercise tolerance, performance and recovery is warranted, particularly in light of metabolomic advances [41]. High dose GTE has been demonstrated to reduce muscle soreness following strenuous exercise [42], potentially via signalling interactions leading to reduced post exercise inflammatory cascades [43]. Results in the current study may have been augmented due to utilisation of a low polyphenol diet. Further research combining dietary polyphenols with dGTE is warranted.

## Conclusions

In conclusion, dGTE in conjunction with exercise training reduced relative FAT_TOT_ and body composition in recreationally active, male volunteers. Improved metabolic efficiency during submaximal exercise may potentiate improved metabolic economy and hence adherence to longer term training programmes. Combined with the observed impact of dGTE on subsequent performance indices, this supports the contention that EGCG use may modulate cellular signalling pathways leading to more efficient substrate use, resulting in improved exercise output.

## Abbreviations

CHO: Carbohydrate
CHO_TOT_: Total carbohydrate oxidation rate (measured in g·min^−1^)
COMT: Catechol-o-methyl transferase
dGTE: Decaffeinated green tea extract
EE: Energy expenditure (measured in kJ·min^−1^)
EGCG: (−)-Epigallocatechin-3-gallate
FAT_TOT_: Total fat oxidation rate (measured in g·min^−1^)
GT: Green tea
PL: Placebo formula used in the study
RER: Respiratory exchange ratio the ratio from dividing expired carbon dioxide with oxygen uptake
RPE: Rating of perceived exertion
TFA: Total fatty acids
$\dot{\mathrm{V}}{\mathrm{O}}_2$: Volume of oxygen uptake (measured in L·min^−1^)
$\dot{\mathrm{V}}{\mathrm{O}}_{2\mathrm{peak}}$: Peak oxygen uptake (measured in L·min^−1^)
$\dot{\mathrm{V}}{\mathrm{CO}}_2$: Volume of expired carbon dioxide (measured in L·min^−1^)
